# Accelerating photonic gas sensor design: machine learning-driven inverse optimization of silicon photonics Bragg gratings

**DOI:** 10.1038/s41598-026-43725-z

**Published:** 2026-04-07

**Authors:** Maira Khafagy, Merna Khafagy, Mohamed A. Swillam

**Affiliations:** Department of Physics, School of Science and Engineering, The American University in Cairo, New Cairo, 11835 Egypt

**Keywords:** Bragg grating, Gas sensing, Deep neural networks, Optuna optimization, Inverse design, Stacking ensemble learning, Engineering, Materials science, Optics and photonics

## Abstract

Optical gas sensors based on photonic structures offer label-free, real-time detection with high sensitivity, but their design optimization remains computationally expensive. We present a hybrid machine learning framework that automates the inverse design of polymer-filled slot Bragg grating (PSBG) sensors for rapid prototyping across multiple target gases. Our approach combines Optuna-tuned artificial neural networks with a multi-model stacking ensemble (gradient boosting, random forest, XGBoost, ridge regression) to predict optimal structural parameters (grating period, depth, ridge width, slot height) from desired spectral characteristics. The framework incorporates domain-informed preprocessing (polynomial feature expansion, physics-based gas encoding) and per-target weighted meta-learners to ensure design fidelity. Validated on 1,045 design samples for CO_2_ and CH_4_ detection, the model achieves R^2^ > 0.99 for all parameters, enabling inverse design without iterative electromagnetic simulations. This work demonstrates the potential of ensemble learning for accelerating photonic device development and supports scalable platform-based sensor design.

## Introduction

Monitoring toxic gases has become a crucial focus for researchers seeking to ensure public health, industrial safety, and environmental protection^1^. Carbon dioxide (CO_2_) and methane (CH_4_) are essential greenhouse gases that contribute significantly to climate change and environmental degradation^2,3^. In recent years, efforts to improve toxic gas detection have increased, leading to more compact and highly sensitive devices. Optical gas sensors based on refractive index (RI) modulation, especially those that utilize photonic technologies, have several benefits, such as being easy to integrate with other portable devices and very sensitive^4,5^.

Silicon photonics has emerged as a promising platform for optical gas sensing, leveraging CMOS fabrication processes. The strong light confinement in silicon-on-insulator (SOI) waveguides enhances the interaction between the optical guided mode and target gas molecules and improves the sensitivity^6^. Slot Bragg grating structures have shown great potential for gas detection. The design of the slot waveguide can improve how the optical guide interacts with the surrounding gas by confining a significant portion of the electromagnetic field within a narrow low-index region known as the slot^7,8^. When integrated with a Bragg grating, this structure allows for strong wavelength-selective reflection, enabling precise monitoring of refractive index changes due to gas absorption^9,10^.

Although silicon photonic sensors demonstrate promising performance. This method can be time-consuming and less scalable, and it may not thoroughly explore the entire design space needed to achieve optimal sensitivity and selectivity. To address these challenges, recent research has turned to machine learning (ML) and deep learning (DL) techniques, which provide data-driven modeling and prediction capabilities. These models can learn complex relationships between structural parameters and sensor responses, thereby accelerating the design process, improving performance predictions, and guiding the development of high-sensitivity gas sensors^11^.

In a study in^12^, the authors introduced a biosensing platform that utilizes waveguide Bragg gratings for the early detection of cancer. This technology takes advantage of the differences in RI between healthy and cancerous cells. The design of the sensor was optimized using a neural network model to classify samples based on the observed spectral shifts. The work in^13^ improved the performance of a fiber Bragg grating glucose sensor by incorporating a deep learning model with attention mechanisms. This method enhanced the accuracy of spectral feature predictions and reduced experimental noise, showcasing the potential of deep learning to refine photonic sensor data. The work in^14^ utilized DL for predicting the shape of eccentric fiber Bragg grating (eFBG) sensors employed in continuum surgical robots. A Siamese architecture based on convolutional neural network (CNN) was developed and optimized using the Hyperband algorithm to extract strain information from noisy spectral data. The study in^15^ investigated data-efficient machine learning models for predicting the characteristics of surface Bragg gratings in semiconductor waveguides. The predictions were based on a limited dataset generated through 3D Finite-Difference Time-Domain (FDTD) simulations. The authors discovered that the XGBoost model outperformed other models, highlighting the potential of lightweight machine learning approaches for inverse photonic design, particularly when generating simulation data is expensive. The work in^16^ presents a flexible fiber Bragg grating (FBG) sensor featuring a spiral structure designed for directional strain sensing. To analyze the irregular spectral patterns induced by transverse stress, a one-dimensional CNN was employed to predict the force-bearing angle. The model demonstrated high accuracy, achieving angle recognition errors of less than ${2}^{o}.$.

Previous studies have demonstrated that ML and DL can enhance silicon photonic sensors by improving spectral interpreta tion, reducing noise, and optimizing shape sensing. However, these approaches have primarily concentrated on processing data after the design phase. The application of artificial intelligence (AI) in the direct design of photonic sensors, especially for gas detection, has not been thoroughly investigated. Designing a sensitive sensor to detect $C{O}_{2}$ and CH_4_ is challenging because the changes in RI caused by low concentrations of these gases are very subtle. Current ML and DL models do not encompass the entire design process needed to customize sensor parameters for specific types of gases. This study proposes a DL-driven inverse design approach, enabling the automated generation of Polymer Phase shift Bragg grating (PPSBG) structures based on the target gas. Our approach begins with polynomial feature expansion and data standardization, followed by an Optuna-tuned artificial neural network (ANN) and multiple tree-based base models. These base learners are combined through stacking with per-target meta-learners to enhance prediction accuracy. A weighted loss function is introduced in the ANN to prioritize specific target parameters, ensuring design fidelity for critical sensor dimensions. It is important to clarify that this work addresses a single and well-defined technical problem: the inverse design of polymer slot Bragg gratings from prescribed spectral features. Although the literature review discusses studies involving classification and sensing tasks, these are included only to highlight the broader role of machine learning in photonic devices. Our contribution does not perform gas classification nor spectral sensing inference; instead, our model assumes the spectral characteristics as given and focuses exclusively on predicting the structural parameters of the PPSBG. We introduce a hybrid stacking ensemble that combines the complementary strengths of neural networks and tree-based learners, whereas prior inverse-design studies typically rely on a single model such as XGBoost or MLPs. Our pipeline incorporates physics-informed preprocessing, including RI-based gas encoding and polynomial expansion—that embeds domain knowledge into the learning process before training. These contributions collectively enable a more accurate, scalable, and physically consistent inverse-design framework for polymer slot Bragg grating gas sensors.

This paper is organized to provide a comprehensive overview of the design as following “Theory and design” describes the theory and design of the proposed PPSBG, including the structural concept and the role of polymer materials in enhancing sensitivity. “Dataset generation” explains the dataset generation process, detailing the relationship between the input and output parameters and the data-splitting strategy. “Data preprocessing” focuses on data preprocessing, including feature expansion, gas variable encoding, and data standardization procedures. “Model architecture” introduces model architecture, discussing the Optuna-tuned ANN, tree-based learners, and the stacking ensemble framework. “Deep learning training model” elaborates on the deep learning training process, covering the weighted loss function, optimization procedure, and final model configuration. Finally, “Results and performance evaluation of ANN and stacking ensemble” presents the results and performance evaluation, highlighting the model’s predictive accuracy and comparing it with previously reported works to demonstrate the efficiency of the proposed inverse-design approach for gas sensing applications.

## Theory and design

### Phase shift bragg grating design for sensing

**Fig. 1:**
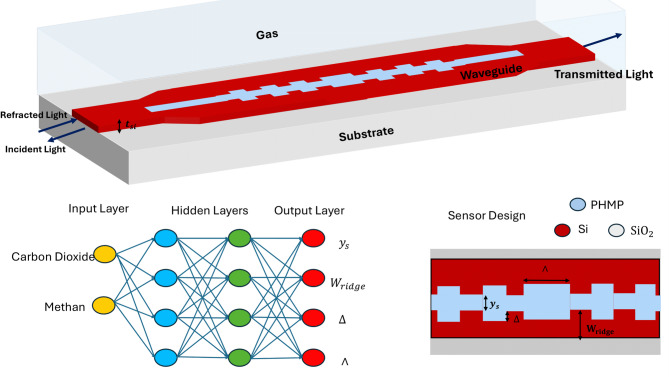
Schematic of the proposed SPSBG sensor.

The schematic of the proposed slotted phase-shifted Bragg grating (SPSBG) structure is shown in Fig. 1. The proposed SPSBG is simulated based on a silicon-on-insulator (SOI) platform, where silicon is the core layer with a thickness of 220 nm mounted on the silicon dioxide substrate. The waveguide has two parallel silicon arms that are separated by a narrow slot filled with polymer material. This slot region enhances light-matter interaction that improves the sensing performance. To form the Bragg grating, corrugations are patterned along the inner sidewalls of the silicon arms.

Light propagates through the slot when the effective RI of the guided optical mode (${n}_{eff}$) satisfies the condition ${n}_{slot}$*<* ${n}_{eff}$ *<* ${n}_{core}$. A higher value of ${n}_{eff}$ results in stronger optical confinement within the slot and improved propagation efficiency. The grating period Λ is designed based on the calculated ${n}_{eff}$ at the operating wavelength to ensure the formation of a strong resonance. The design follows the Bragg condition:

1 $${\lambda}_{B}=2{n}_{eff}\varLambda$$

where ${\lambda}_{B}$ is the Bragg wavelength. A central phase-shift region is introduced by locally modifying the grating pattern, which sharpens the resonance and enhances the spectral response. The grating segments are symmetrically distributed on both sides of this phase-shift defect, leading to strong field confinement and higher sensitivity.

In addition to illustrating the physical geometry, Fig. 1 defines the key structural parameters employed in the inverse-design framework: the grating period (Λ), modulation depth (Δ), ridge width (W), and slot height (H). These geometrical variables directly determine the effective refractive index and govern the Bragg resonance condition. Accordingly, they serve as the regression targets of the proposed neural network model. Establishing this explicit correspondence between the device geometry and the learning outputs clarifies how the physical SPSBG structure is translated into a supervised inverse-design problem.

### Polymer material for sensitivity enhancement

The integration of functional polymer materials into photonic structures offers a powerful approach to enhancing the sensitivity and selectivity of optical gas sensors. In the context of phase-shifted Bragg grating (PSBG) designs, these polymers are introduced into the slot region of a silicon waveguide, where their refractive index properties can be exploited to modulate the optical response of the device in the presence of target gases. The underlying sensing mechanism is based on the interaction between the gas molecules and the polymer, which leads to changes in the polymer’s refractive index and, in some cases, its physical dimensions due to swelling. These changes will affect the effectiveness of the RI (${n}_{eff}$) of the guided optical mode, thereby shifting the Bragg resonance condition.

A wide variety of polymers are available for gas sensing applications, each exhibiting unique affinities toward specific analytes. This selectivity arises from the chemical compatibility between the gas molecules and the functional groups within the polymer chains. For instance, poly (hexamethylene biguanide) (PHMB) exhibits a strong interaction with carbon dioxide ($C{O}_{2}$), making it an effective sensing material for CO_2_ detection. Similarly, polymethyl methacrylate (PMMA) shows good sensitivity to methane ($C{H}_{4}$) due to its favorable absorption and swelling characteristics when exposed to hydrocarbon gases. These polymer-gas interactions cause a measurable shift in the Bragg wavelength, enabling label-free, real-time detection with high resolution.

The incorporation of polymers into the slotted waveguide geometry enables the optical field to be confined within the low-RI slot region. This configuration ensures that the guided mode overlaps the polymer material, enhancing the light–matter interaction and leading to increased sensitivity of the device. Compared to conventional ridge or strip waveguides, slotted structures provide a higher fraction of the optical mode within the analyte-sensitive region, resulting in improved sensor performance.

In this work, the use of polymer materials is not only critical for gas-specific detection but also enables a modular approach to sensor design. By selecting different polymers for different target gases, the sensing platform can be rapidly reconfigured to detect various environmental or industrial gases. Furthermore, the integration of an artificial neural network (ANN) allows for rapid inverse design of the Bragg grating parameters based on the chosen polymer’s refractive index and the desired spectral response. This approach streamlines the design process and supports the development of highly sensitive, application-specific photonic gas sensors.

## Dataset generation

The dataset utilized in this study comprises a combination of simulated results and numerical data processing, which represents the inverse design mapping of the proposed SPSBG gas sensor. The aim of this dataset is to establish a supervised learning framework in which spectral responses function as the input features, while the geometrical and structural parameters of the sensor act as the output targets.

### Input features and output predictions

The input nodes of the proposed SPSBG sensor are defined by four key features that characterize its spectral response. These include the Gas, a categorical variable representing the target analyte that is numerically encoded to reflect gas-induced changes in the refractive index of the polymer cladding; the Transmission Amplitude, which denotes the normalized transmission intensity at the resonance wavelength; the Center Wavelength, indicating the spectral position of the resonance peak; and the Full Width at Half Maximum (FWHM), which represents the spectral linewidth and corresponds to the sensing resolution of the device. By capturing these spectral signatures, the dataset effectively encodes the physical differences among multiple gases.

**Fig. 2:**
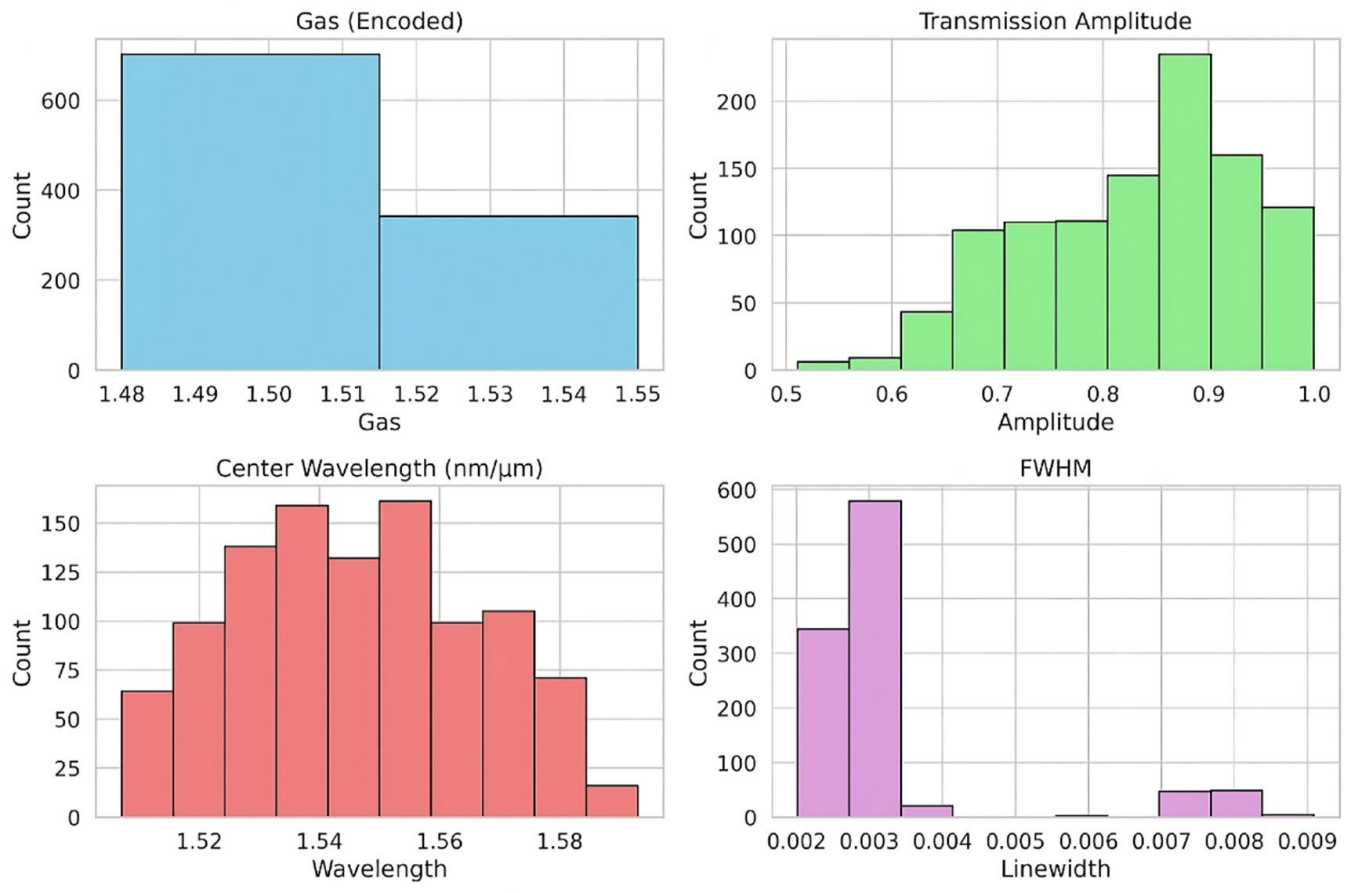
Histograms of dataset features: (**a**) gas, (**b**) transmission amplitude, (**c**) center wavelength, and (**d**) FWHM.

The statistical distribution of the dataset features is illustrated in Fig. 2, which includes histograms for gas identity encoding, transmission amplitude, center wavelength, and FWHM, These parameters are clearly defined and physically explained in[7] The gas encoding reveals an imbalanced distribution, with one gas type being more prevalent than the others, which may impact the model’s ability to generalize. The transmission amplitudes primarily range from 0.7 to 0.95, indicating that most samples demonstrate high transmission characteristics. The center wavelength appears to follow a uniform distribution between 1.51 *µ*m and 1.58 *µ*m, providing good coverage of the design space. In contrast, the FWHM distribution is highly skewed, with most samples clustered between 0.002 and 0.003. This reflects the narrow linewidth behavior that is characteristic of high-quality optical resonances. Within this machine-learning framework, the geometric parameters of the SPSBG—namely the grating period Λ, modulation depth *δ*, ridge width ${W}_{ridge}$, and slot width ${y}_{s}$ —serve as the model outputs. This arrangement enables automated inverse design: given any target spectrum (defined by the gas type and its corresponding spectral characteristics), the model directly predicts the structural parameters required to generate that response. In our work, the dataset was constructed based on the design boundaries reported in previous studies^7^. Using this prior knowledge, we performed systematic parameter sweeps in which one structural parameter was varied at a time while the others were held constant. As expected, the phase-shifted resonance begins to degrade when operating at or beyond these boundaries, confirming that the selected parameter limits are consistent with the physical behavior of the device. Table 1 summarizes the full range of all geometric parameters included in the dataset and describes the effect of each parameter on the transmission spectrum. This analysis was conducted in detail in our previous work. These ranges define the practical design space in which the ANN model was trained and validated. To further expand the dataset or explore new design regions, a multidimensional sweep—varying all parameters simultaneously—can be conducted. This would allow the discovery of additional parameter combinations, new feasible boundaries, and potentially improved sensing configurations. Such higher-dimensional sampling can be incorporated in future work to broaden the model’s generalization capability.

**Table 1:** Unified parameter ranges for CO2 and CH4 datasets.

Parameter	Range (CO2 / CH4) [µm]	Impact
Period	0.45–0.50 / 0.38–0.48	Bragg condition
Depth	0.01–0.02	Grating confinement
Ridge width	0.22–0.25	Field overlap
Slot height	0.09–0.10	Light–matter interaction

### Correlation analysis between input and output

This subsection analyzes the correlations between the input spectral features and the output structural parameters of the proposed SPSBG sensor. The degree of linear relationship between variables is measured using Pearson’s correlation coefficient (*/r*). Values near + 1 reflect a strong positive correlation, values near *−* 1 reflect a strong negative correlation, and minimal linear dependency is suggested by values near 0.

**Fig. 3:**
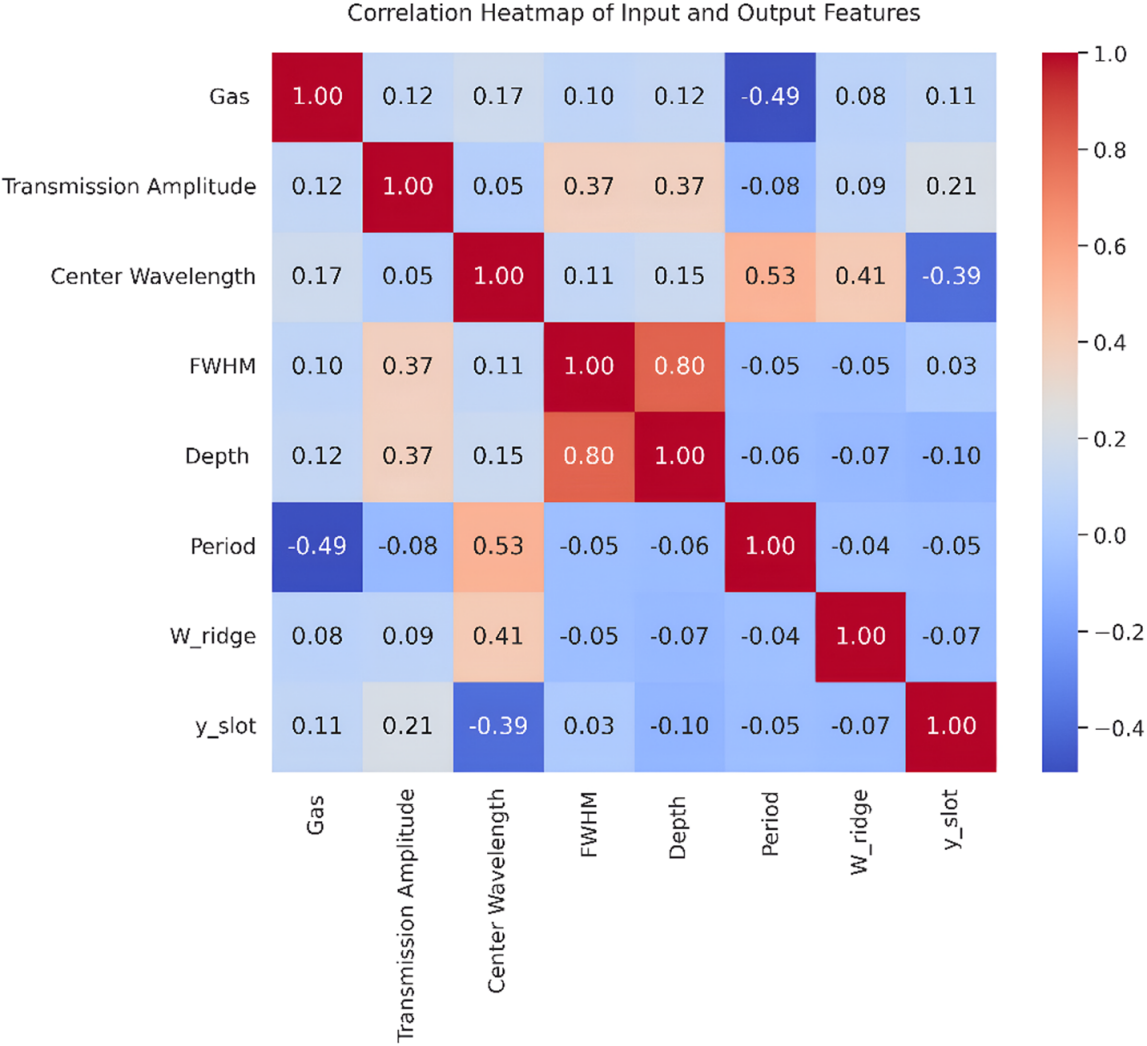
Correlation heatmap of input and output features for the inverse design of the proposed SPSBG sensor.

The correlation heatmap, shown in Fig. 3, provides an overview of the interdependencies within the dataset. The slot Depth shows a strong positive correlation with FWHM (*r* = 0.80), indicating that deeper corrugations lead to a broader resonance linewidth. Additionally, the grating Period shows a moderate positive correlation with the Center Wavelength (*r* = 0.53), which aligns with the fundamental physical principle of Bragg gratings, where the periodicity directly influences the resonant wavelength. In contrast, the encoded Gas type exhibits a moderate negative correlation with Period (*r* = − 0.49), suggesting that the target gas analyte, through its effect on the refractive index of the polymer cladding, influences the optimal grating period required to achieve the target resonance.

### Dataset splitting

**Fig. 4:**
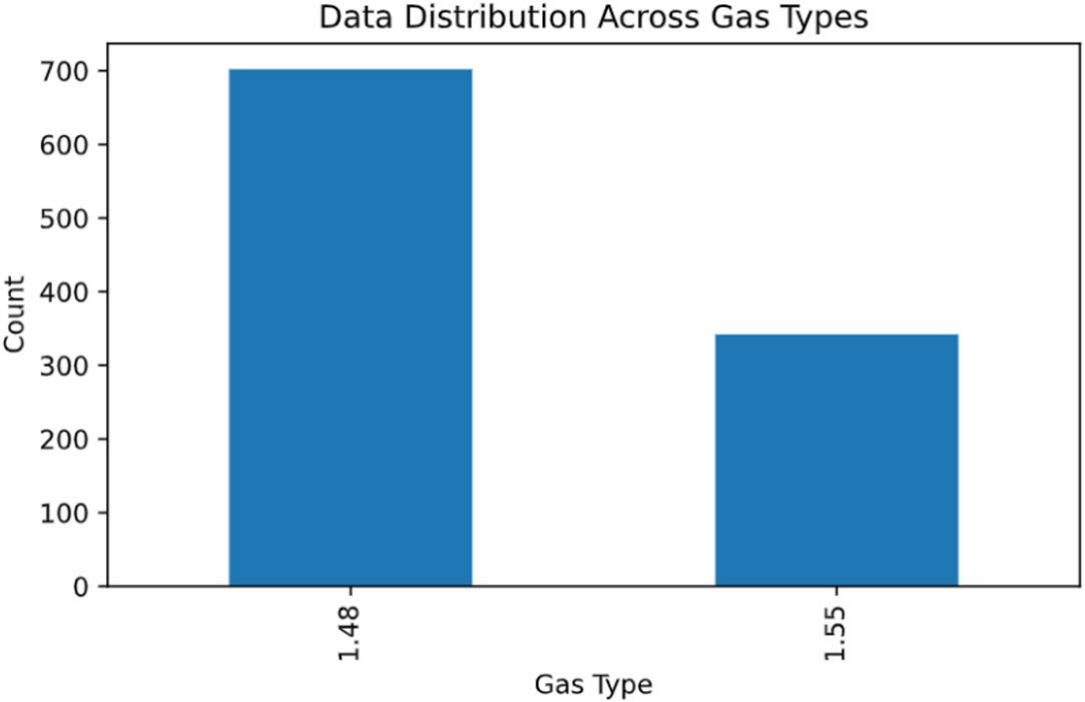
Data distribution across gas types and design space regions.

The total dataset comprises 1045 distinct designs of the proposed PPSBG sensor, which collectively form the foundation for training and evaluating the deep learning model. Figure 4 shows different gas categories used for model development, the distribution of data points for each gas type was examined. The resulting histogram demonstrates a clear variation in the number of measurements per class, where some gas types include a noticeably larger sample count compared to others. This imbalance highlights the importance of applying appropriate validation and training strategies to avoid bias toward the more represented classes.

To assess the model’s performance, the dataset was divided into two subsets: a training set and a testing set. An 80/20 split was adopted, allocating 80% (836 designs) for training—used to optimize and fine-tune the model’s internal parameters—and 20% (209 designs) for independent testing. This division ensures that the model is exposed to a sufficiently diverse range of input–output mappings during training. Meanwhile, the test set provides an unbiased evaluation of the model’s ability to generalize and accurately predict unseen data. The data splitting was performed randomly yet consistently across all experiments to ensure reproducibility and fairness in the evaluation process.

###  Feature representation across gas types

**Fig. 5:**
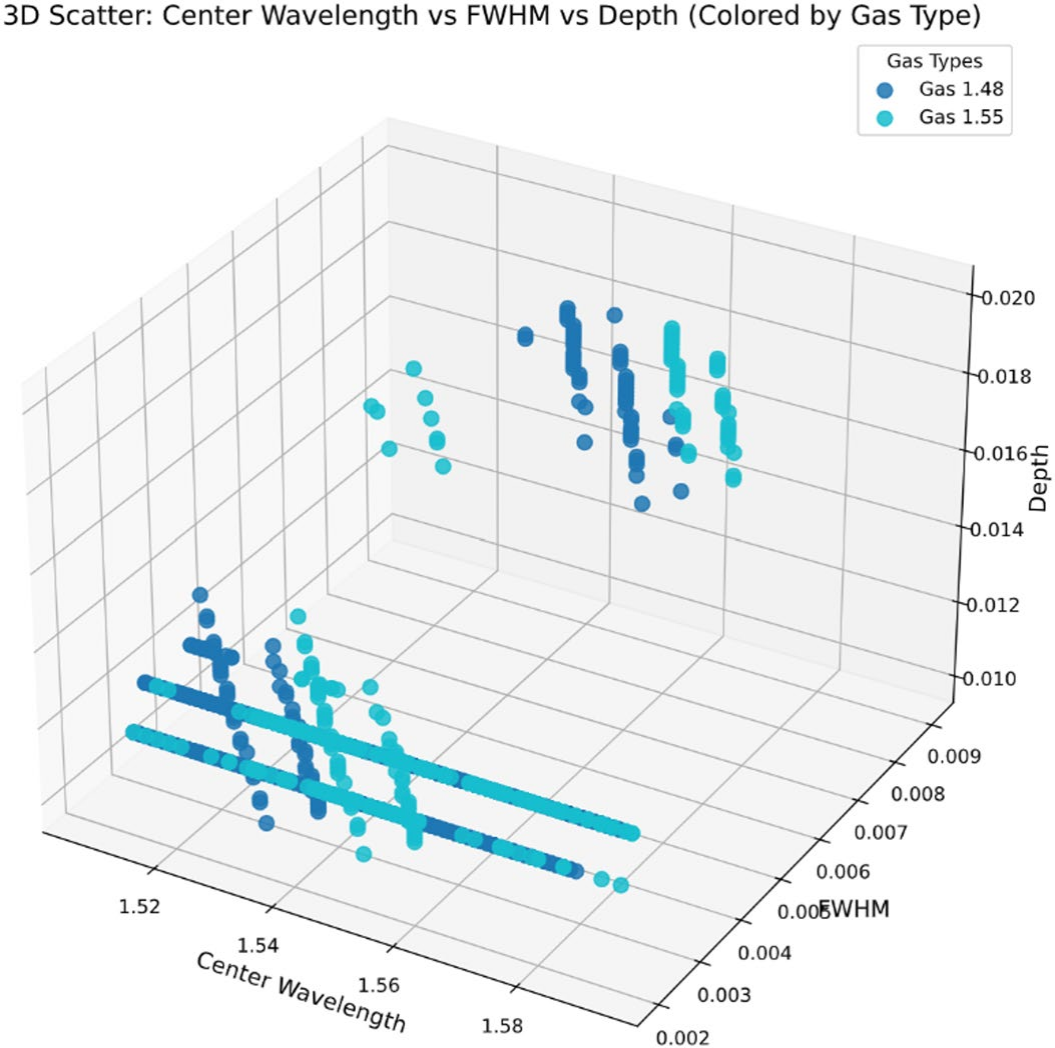
Three-dimensional scatter plot of center wavelength, FWHM, and depth, with points color-coded according to gas type.

To represent the dataset’s structure and assess the model’s ability to generalize, Fig. 5 shows a three-dimensional scatter plot with Center Wavelength, FWHM, and Depth as axes. The visualization clearly shows two distinct clusters representing the two encoded gases (Gas 1.48 and Gas 1.55), indicating that each gas displays a different physical response within this feature space. The Depth parameter generates a smooth and continuous surface concerning both the Center Wavelength and the FWHM. This highlights the meaningful correlations among the variables. The absence of outliers or irregular patterns indicates that the dataset is well-structured and suitable for supervised learning.

##  Data preprocessing

**Fig. 6:**
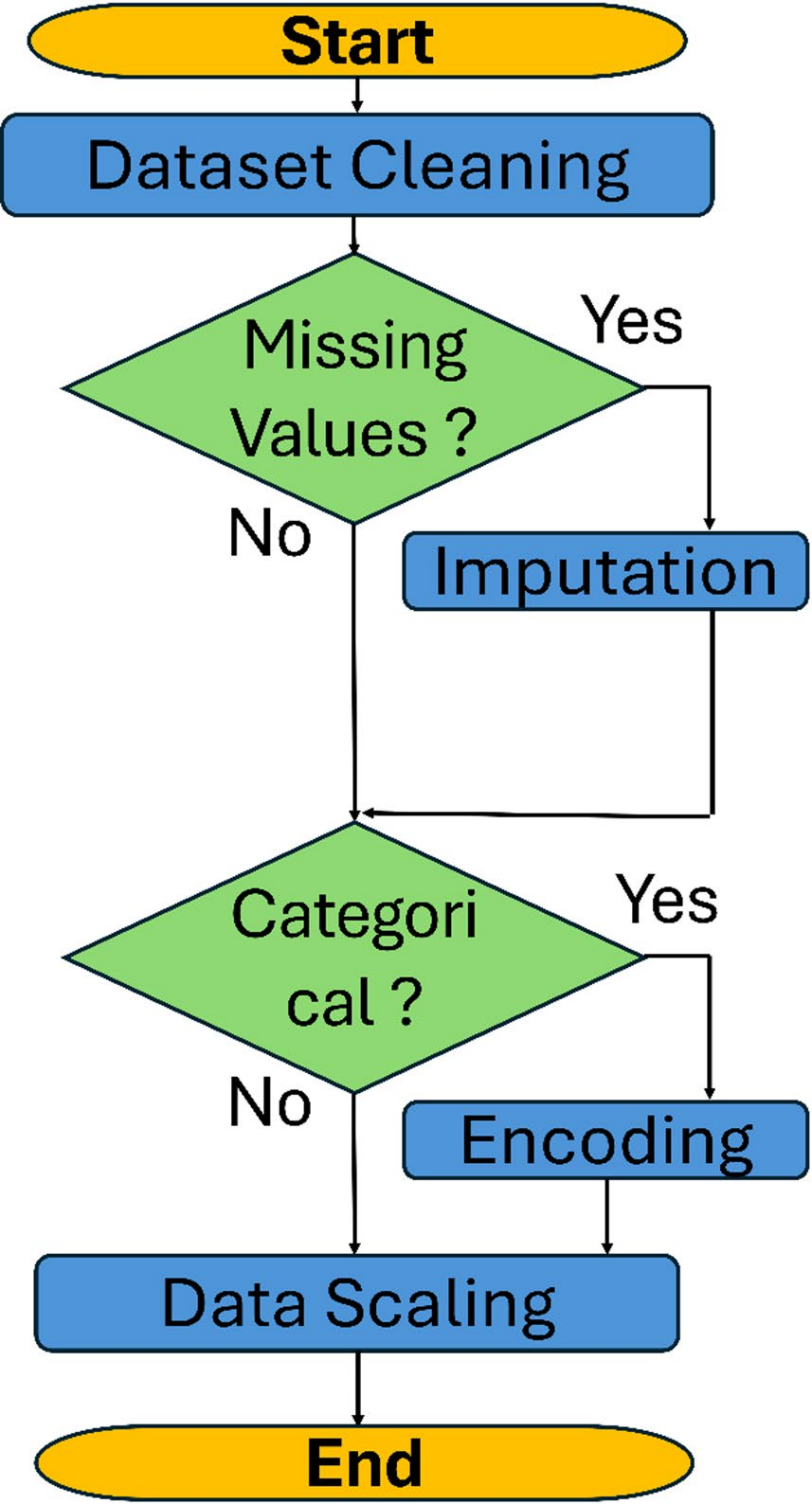
Flowchart of the data preprocessing pipeline.

Data preprocessing is an important stage in this study, ensuring that the dataset is properly prepared before it is fed into the DNN model. The flowchart in Fig. 6 illustrates the preprocessing pipeline utilized for our dataset. First, the dataset is cleaned to address any missing values^17^. Next, we perform polynomial feature expansion to capture higher-order relationships for the input variables. Following this, data scaling is applied to standardize the feature space and prevent bias arising from differences in magnitude^18^. Finally, we utilize an encoding technique for categorical variables, such as gas type.

### Polynomial feature expansion

The proposed PPSBG utilizes a nonlinear relationship between the input spectral features and the output parameter, which produces challenges for design, as the small variations in the transmission amplitude may produce non-proportional changes in the ∆, while the combined effect of gas RI and center wavelength can jointly shift the Bragg resonance. Traditional linear models fail to capture such intricate dependencies, motivating the introduction of polynomial feature expansion to enrich the feature space with higher-order interactions. Formally, for an input vector^19^

2 $$x=\left[{x}_{1}{x}_{2}{x}_{3}{x}_{4}\right]$$

where ${x}_{1}$denotes the encoded gas type, ${x}_{2}$ the transmission amplitude, ${x}_{3}$the center wavelength, and ${x}_{4}$the FWHM, the polynomial mapping up to degree three is defined as

3 $$\varPhi\left(x\right)=\left\{{x}_{i},{x}_{i}^{2},{x}_{i}{x}_{j},{x}_{i}^{3},{x}_{ij}^{2},{x}_{i}{x}_{j}{x}_{k},...\right\}fori,j,k\in\left\{\mathrm{1,2},\mathrm{3,4}\right\}$$

This expansion includes linear, quadratic, cubic, and interaction terms. The transformation guarantees that all squared terms, cubic terms, and cross-terms are maintained, while a constant bias term is excluded. As a result, the original four input variables are expanded to a total of 34 characteristics: 4 linear terms, 10 quadratic terms, and 20 cubic terms. This increase in dimensionality offers learning models a deeper understanding of the underlying physics. By introducing polynomial features, the proposed pipeline allows predictive models to better approximate nonlinear relationships. Cross-terms, such as the product of wavelength and amplitude, help capture the interdependent relationships between spectral variables.

### Encoding the gas variable

Among the four input variables considered in this study, the Gas parameter is categorical in nature, as it represents different analytes ( $\mathrm{C}{\mathrm{O}}_{2}$and $\mathrm{C}{\mathrm{H}}_{4}$) to be detected by the PPSBG. The DNN models, especially those using numerical optimization, cannot directly process categorical labels; thus, these variables require numerical encoding. To overcome this limitation, an encoding strategy is required to translate categorical labels into numerical representations that can be processed by the network^20^.

In this study, we first mapped each gas type to its corresponding RI value in the near-infrared spectrum. The $\mathrm{C}{\mathrm{O}}_{2}$ was assigned an RI of 1.55, while CH_4_ was represented by a 1.48. Unlike raw label encoding, which arbitrarily assigns integers $\mathrm{C}{\mathrm{O}}_{2}$ = 1, $\mathrm{C}{\mathrm{H}}_{4}$ = 2 without physical meaning, the RI-based encoding embeds domain knowledge into the dataset. This RI-based encoding allows us to transform the categorical variable into a numerical format that retains physical meaning within the dataset. Although one-hot encoding could also be used—representing gases as orthogonal binary vectors (e.g., $\mathrm{C}{\mathrm{O}}_{2}$ = [1,0], $\mathrm{C}{\mathrm{H}}_{4}$ = [0,1])—this method ignores the underlying physics. By contrast, RI encoding integrates seamlessly with the other numerical inputs (transmission amplitude, center wavelength, FWHM) and enables polynomial expansion for capturing nonlinear spectral interactions.

### Feature and target standardization

The performance of both neural networks and tree-based algorithms can vary depending on the scaling of their input features. When features have significantly different ranges, those with larger magnitudes can dominate the learning process. This can result in biased weight updates and slower convergence. To mitigate this issue, all input features in this study were standardized before training. To address this, both input features and target variables were standardized. The standardization transformation rescales each variable to have zero mean and unit variance, according to^21^

4 $${x}^{{\prime}}=\frac{x-\mu}{\sigma}$$

where *σ* is the standard deviation and *µ* is the mean of each feature or target. The same transformation was applied independently to all input features and all output variables.

## Model architecture

The proposed deep learning model for inverse design utilizes a stacked learning strategy. In this approach, multiple base models are trained to capture different aspects of data distribution. Their predictions are then combined through meta-learners to achieve a high result. This design allows to address the nonlinear and multi-output nature of the problem, with the objective of mapping spectral and gas-related features to the structural parameters of the polymer slot Bragg grating.

###  Comparison of learning-based and optimization-based baselines

The first step in this study is to evaluate different models to establish a reference level of performance. We test several simple baselines that are widely used in photonic inverse design. The first model is a baseline ANN, implemented as a feedforward neural network with a few dense layers trained using mean squared error loss. This ANN serves as the simplest learning-based model capable of mapping spectral inputs to structural outputs. The second model is the Genetic Algorithm (GA), a classical optimization method frequently used in photonic design. Instead of learning a functional mapping, GA searches for a single set of structural parameters that minimizes the global error over the entire dataset. Finally, Simulated Annealing (SA) is included as an additional optimization baseline. SA is another widely used heuristic search method that attempts to escape local minima using probabilistic acceptance rules during a gradual “cooling” process. Similar to GA, SA does not learn sample-specific relationships and instead attempts to optimize one global design. These baselines allow us to directly compare learning-based regression with classical optimization-based design and assess whether neural networks offer clear advantages for this inverse-design problem. The simple ANN baseline achieved an overall *R*^2^ = 0.6898, demonstrating moderate predictive despite the absence of hyperparameter tuning, residual connections, or weighting strategies. In contrast, the GA baseline achieved *R*^2^ = − 0.0130, indicating that it fails to generalize and cannot approximate the required multi-parameter inverse mapping. Simulated Annealing performed even worse, with *R*^2^ = − 0.4055, confirming that SA is also unable to capture the nonlinear, input-dependent relationships between the spectral features and structural parameters.

### Gradient-based optimization methods

Automated gradient-based methods, such as Lumerical’s built-in adjoint optimizer and the SPINS inverse-design framework, represent powerful tools for physics-driven photonic optimization. These techniques update structural parameters by computing sensitivity gradients, enabling rapid improvement of a single device design through iterative simulation feedback. While such approaches are highly efficient for optimizing an individual geometry, they are not suitable for learning a global inverse mapping from spectra to structural parameters.

Gradient-based physics solvers are fundamentally single-instance optimizers: each new target spectrum requires a full optimization run, often involving hundreds of forward and adjoint simulations. Moreover, these methods are sensitive to initialization, may become trapped in local minima, and do not generalize across samples. In contrast, the ANN-based approach developed in this work learns a dataset-level functional relationship that produces predictions in milliseconds after training. This makes neural networks significantly more scalable for multi-output inverse design problems. For this reason, gradient-based optimization methods are discussed as related techniques but are not directly comparable to data-driven inverse models such as ANN, GA, SA, or the proposed ensemble framework.

### Optuna-tuned artificial neural network (ANN)

The ANNs are powerful models for nonlinear relationships between input and output tasks. However, their effectiveness is influenced by the chosen hyperparameters, including the number of hidden layers, the number of neurons in each layer, the activation functions, and the learning rate. To overcome the challenge of manual tuning, we employed Optuna, an automatic hyperparameter optimization framework based on Bayesian optimization and pruning strategies.

Optuna uses a define-by-run interface, enabling flexible construction of the search space during the optimization process. It uses an efficient Bayesian optimization strategy, particularly the Tree-structured Parzen Estimator (TPE), which models the probability distributions of good and bad hyperparameter regions. By adaptively managing the exploration-exploitation trade-off, Optuna achieves superior sampling efficiency. This results in a marked reduction in the computational resources required to identify optimal hyperparameters when benchmarked against conventional methods like grid and random search.

Each trial performed by Optuna represents a single candidate configuration of hyperparameters. The training and validation performance (measured using the chosen evaluation metric, e.g., accuracy or F1-score) served as the objective function to be minimized/maximized. Optuna employs pruning techniques, which allow early termination of unpromising trials based on intermediate results, further accelerating the optimization process.

Formally, the optimization problem can be written as^22^:

5 $${\theta}^{*}={}_{\theta\in\varTheta}^{argM{a}_{x.}M\left(\theta\right)}$$

where *θ*, denotes the set of hyperparameters, Θ is the defined search space, and *M*(*θ*) represents the performance of the model under the configuration *θ*. After conducting *N* tests, Optuna returned the best hyperparameter configuration, which was subsequently used to retrain the final model in the training data set.

### Tree-based and linear learners

- Gradient boosting (GB): creates a sequence of weak learners—typically shallow decision trees—where each subsequent tree is trained to correct the residual errors of the previous ones by following the negative gradient of the loss. This iterative refinement process enables the ensemble to capture nonlinear patterns.
- Random forest (RF): is an ensemble approach that combines the outputs of many decision trees trained simultaneously. Each tree is built from a bootstrapped version of the dataset and selects a random set of features at each split, increasing variability across the trees.
- Extreme gradient boosting (XGBoot): it incorporates built-in regularization (both *ℓ*_1_ and *ℓ*_2_) to prevent overfitting, sparsity-aware splitting to handle missing values, and parallelized training to accelerate computation. Because of its characteristics, XGBoost has become a top method for structured tabular data in predictive modeling competitions and practical uses.
- Ridge regression: this model serves as a linear baseline by extending ordinary least squares regression with an *ℓ*_2_ penalty on the magnitude of coefficients. This regularization shrinks less informative weights towards zero, mitigating multicollinearity and preventing overfitting, especially in high-dimensional settings.

### Stacking strategy

To improve the accuracy of the base models, we used a stacking approach. A most important step in stacking is generating out-of-fold (OOF) predictions, which serve as clean and unbiased inputs for training the final meta-learner. In our study, OOF predictions were created using 5-fold cross-validation. The dataset was randomly shuffled and split into five equal parts using a K-Fold method with a fixed random seed to ensure the folds. In each fold, four subsets were utilized to train a specific base model, while the remaining subset was used as the validation fold to generate predictions. This process was repeated five times to ensure that each sample appeared once in the OOF validation set. The OOF predictions produced by each base learner for all four target parameters were then concatenated to form the meta-feature matrix. The Meta-feature formation represents the base learner *k* and each target *m*, which we collect the out-of-fold predictions into a meta-feature matrix:

6 $$Z={y}_{\left(1\right)}^{1:m},{y}_{\left(2\right)}^{1:m},\dots{y}_{\left(k\right)}^{1:m}$$

where *k* denotes the number of base models, and *m* denotes the number of output targets. Each column block corresponds to the OOF predictions of a particular base learner across the 5-fold CV. Because these predictions are generated on unseen folds, the resulting matrix provides a non-leaking, unbiased representation of base-model performance. This matrix constitutes the input to the meta-learner, which is trained afterward on the complete set of OOF meta-features. This explicit OOF-based stacking procedure ensures that (i) no training sample is ever used to generate its own meta-feature and (ii) the meta-learner receives clean, informative inputs, ultimately improving generalization and preventing overfitting.

In this study, we train a separate meta-learner for each target parameter, which helps capture task-specific relationships. We primarily use Ridge regression and ANN as meta-learners because they effectively balance interpretability with nonlinear modeling capabilities. Unlike standard multi-output regression (single meta-learner for all targets), our per-target approach allows each structural parameter to receive specialized optimization. This is justified because: (1) grating Period primarily determines resonance wavelength (physical decoupling from Ridge Width), (2) Slot Height governs sensitivity but weakly couples to Period (soft interdependency), (3) correlation analysis Fig. 3 shows moderate interdependencies (*r <* 0.6), supporting separate treatment. In the ablation study presented in Sect.  4, the ANN meta-learner achieved an *R*^2^ value of 0.6898 when using a single global model, whereas incorporating the per-target base learner improved the performance significantly, yielding an *R*^2^ is 0.99, confirms *≈* 0.3% *R*^2^ improvement from stratification. Since not all parameters equally influence the optical response of the Bragg grating, we introduce a weighted mean squared error (WMSE) to highlight the most important structural parameters, such as the Period and Slot Height. The loss function is defined as follows:

7 $$l=\sum_{i=1}^{n}{w}_{i}{\left({y}_{i}-\widehat{{y}_{i}}\right)}^{2}$$

where ${y}_{i}$ is the true of the *i*-th parameter while $\widehat{{y}_{i}}$ is the predicted value, and *w*_*i*_ is the weight assigned to that parameter. By assigning larger weights to parameters with greater influence on the Bragg grating’s resonance behavior, the model is guided to prioritize accuracy where it is most impactful.

Table 2 summarizes the weighting scheme applied in this work.

**Table 2:** Weighting scheme for structural parameters in the stacked ANN meta-learner.

Parameter	Weight (${w}_{i}$)
Depth	1
Period	2
Ridge width	1
Slot height	2

**Fig. 7:**
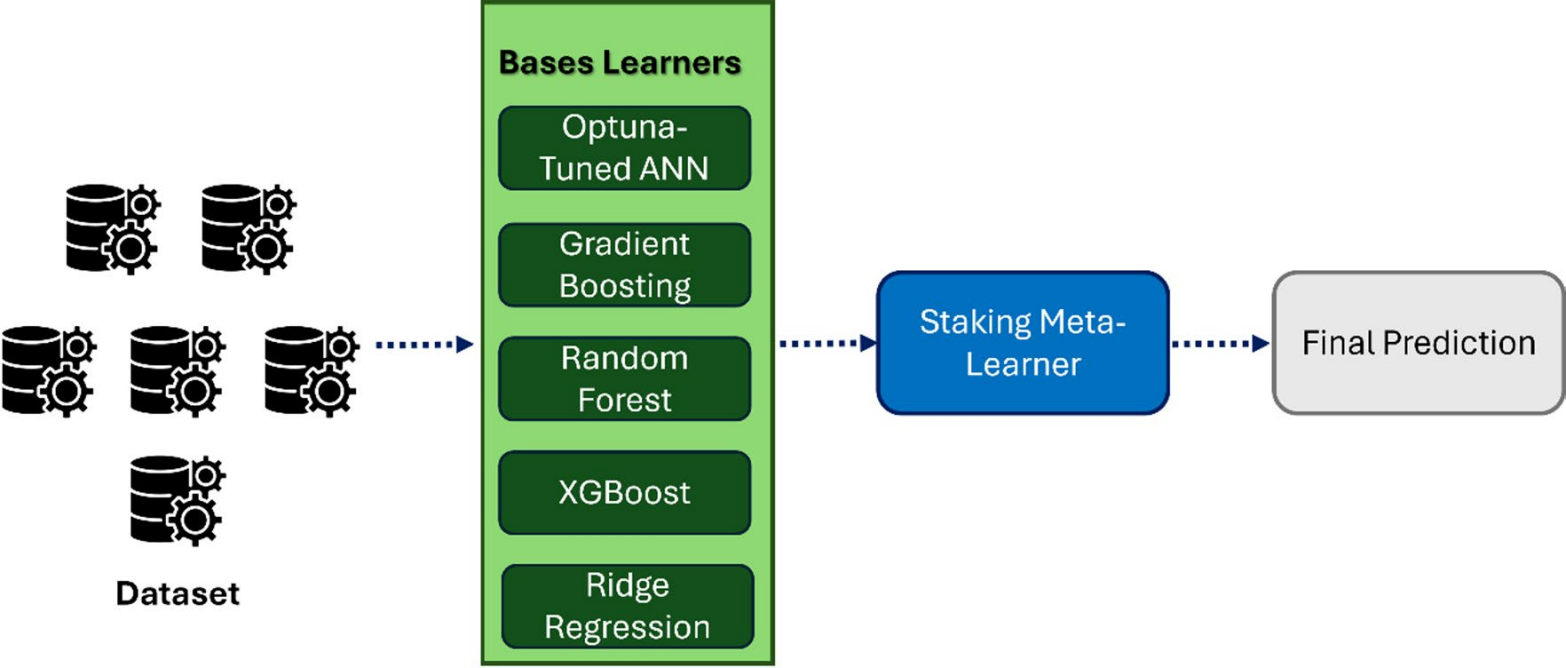
Overview of the stacking ensemble framework.

A schematic of the stacking workflow is illustrated in Fig. 7, showing the two-stage process: base learner predictions.

feeding into the meta-learner, which then outputs the final optimized parameter predictions.

## Deep learning training model

**Fig. 8:**
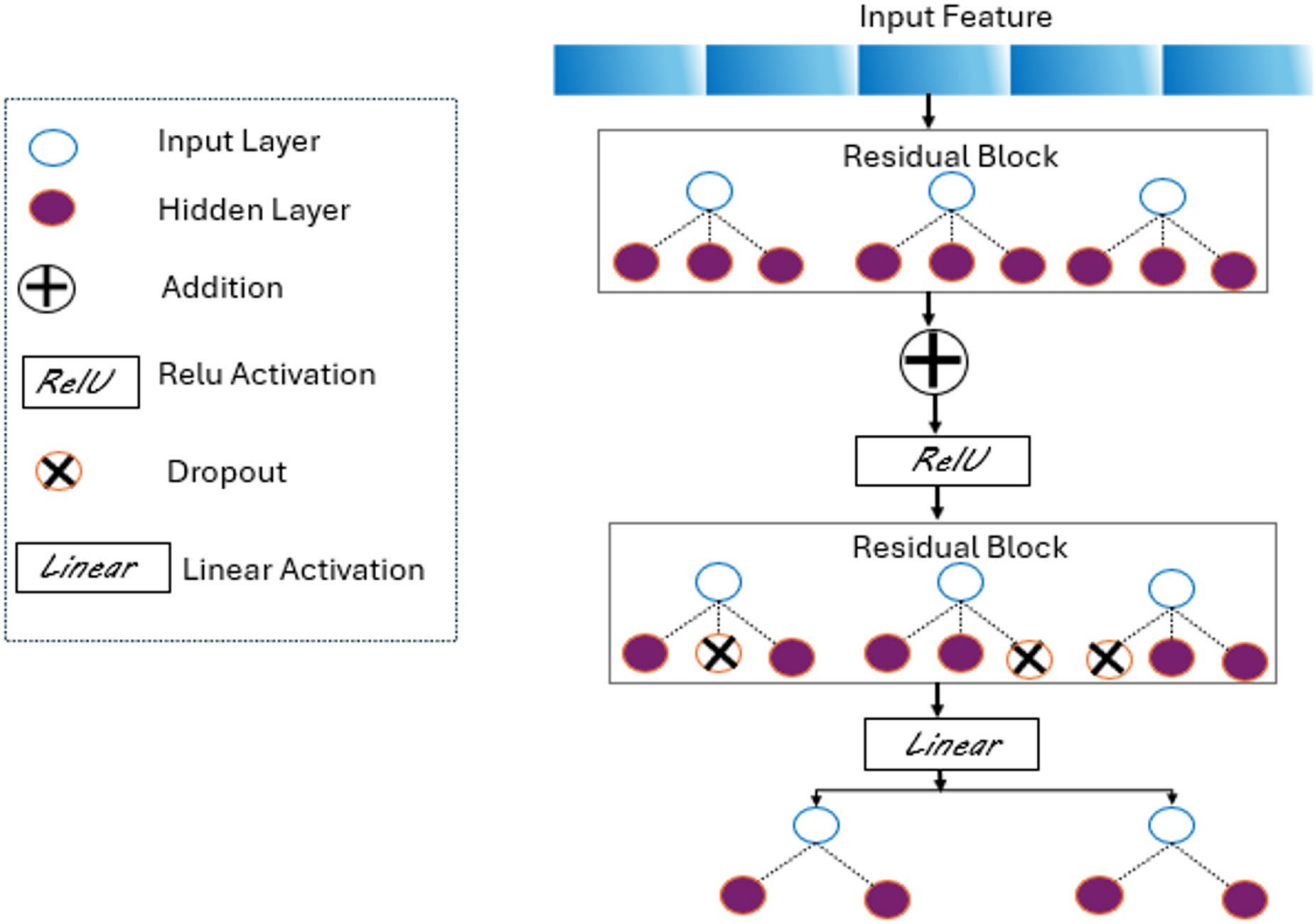
A schematic diagram illustrating the structure of the proposed deep learning model.

The deep learning architecture adopted in this work is schematically illustrated in Fig. 8. The model is based on a fully connected feed-forward neural network augmented with residual connections to facilitate gradient flow and improve convergence stability. The input layer receives the expanded spectral features and feeds them into successive hidden layers organized as residual blocks. Each residual block comprises dense layers followed by batch normalization, ReLU activation functions, and optional dropout units for regularization. The outputs of each block are added element-wise to the block’s input, implementing a skip connection that eases the training of deeper networks. The final layer applies a linear activation to generate continuous predictions for the target parameters.

###  Weighted loss function design

To improve the predictive accuracy of the model, a customized loss function called weighted mean squared error (WMSE) was used during the training of the neural network. The standard MSE assumes that all targets are equally important. However, the sensitivity characteristics of the PPSBG structure show that some parameters have a much stronger impact on the optical response. Therefore, we used a weighting strategy that takes this into account based on physical insights. The explanation behind the weighting scheme is based on physical sensitivity analysis and statistical indicators derived from data. The correlation map in Fig. 3 indicates that Λ and ${y}_{s}$show the strongest linear correlation with spectral features, particularly the center wavelength and FWHM. In PPSBG structures, even minor changes in the two parameters can lead to shifts in the Bragg resonance because of their direct impact on the effective RI and coupling coefficient. In contrast, the variations in ∆ and ${W}_{ridge}$ show weaker correlations and have a lesser influence on the spectrum, making them less critical for resonance alignment.

To translate these physical relationships into the loss function, we assigned a weight vector of (1.0,1.3,1.5,1.3) to the parameters (∆, Λ, ${W}_{ridge}$, ${y}_{s}$), respectively. These weights were determined by first normalizing the relative sensitivities derived from the correlation magnitudes and then validating them through exploratory training runs. In early experiments using an unweighted MSE, the network consistently underperformed in predicting Λ and ${y}_{s}$), resulting in larger prediction variances and poorer spectral matching. By incorporating the weight vector, we increased the gradient contribution of these high-impact parameters, allowing the ANN to focus more of its learning capacity on areas where precision was most critical.

8 $$LWMSE=\frac{1}{N}[\frac{1}{M}\sum_{j=1}^{M}{w}_{j}{\left({y}_{i,j}-\widehat{{y}_{i,j}}\right)}^{2}$$

where, *N* is the number of training samples, *M* is the number of output targets, (${y}_{i,j}$ and $\widehat{{y}_{i,j}}$) and ${w}_{j}$denotes the pre-assigned weight for the *j*-th output variable. Specifically, Λ and ${y}_{slot}$) showed significant reductions in MAE during validation, ultimately enhancing the high spectral accuracy demonstrated in Sect.  6.

#### Physics-based and first-order sensitivity of the weighting scheme

The weighting vector used in the WMSE loss function was derived from a combination of first-order sensitivity analysis and photonic design principles. The first-order sensitivity of each parameter was computed using^7^

9 $${S}_{p}=\frac{\varDelta f}{\varDelta p}$$

where *f* represents spectral features such as the Bragg wavelength or FWHM. Changing Λ and ${y}_{s}$by only *±* 1% caused the largest shifts in the spectrum, which shows that these two parameters strongly affect the device. Λ controls the Bragg wavelength directly through the relation ${\lambda}_{B}=2{n}_{eff}\varLambda$, so even small changes in Λ move the reflection peak. In the same way, changing${y}_{slot}$ modifies how much the optical field is confined inside the polymer-filled slot, which changes the effective RI and therefore shifts *λ*_*B*_ as well. On the other hand, ${W}_{ridge}$ and ∆ caused much smaller changes in the spectrum. This means they have less influence on the device compared to Λ and ${y}_{s}.$For this reason, Λ and ${y}_{s}$were given higher weights, while ${W}_{ridge}$and ∆ were given lower weights. The final normalized weighting vector (1.0, 1.3, 1.5, 1.3) follows this idea and helps the training process focus more on the parameters that affect the sensor performance the most.

#### Rationale for the selected weight vector

In this work, we evaluated three different weighting schemes for the multi-output regression model: uniform weights (1,1,1,1) (or no weights, all outputs treated equally), moderately unbalanced weights (1,2,1,2), and our proposed physically motivated weights (1.0,1.3,1.5,1.3) as summarized in Table 3. The goal of assigning different weights is to emphasize the structural parameters that have the strongest influence on device optical behavior. Among all tested configurations, the proposed weighting scheme consistently provided the most balanced and stable prediction performance across all four targets. In particular, the higher weights assigned to Λ and ${W}_{ridge}$ improved the optimization of these geometric parameters without degrading the accuracy of the remaining targets. Compared with the other tested weighting schemes, our configuration achieved similar or slightly higher *R*^2^ scores while significantly reducing the prediction error for the parameters that dominate the device response. Therefore, the weighted loss using (1.0,1.3,1.5,1.3) provides the best trade-off between overall accuracy and parameter-specific importance, making it the most appropriate choice for physically meaningful inverse design.

**Table 3:** Performance comparison of the ANN + meta-learners under different output weighting schemes.

Parameter	Target	*R* ^2^	MSE	MAE
4*(1, 2, 1, 2)	Depth	0.999651	1.1* × *10^*−* 9^	1.386* × *10^*−* 5^
	Period	0.995481	3.404* × *10^*−* 7^	3.117* × *10^*−* 4^
	${W}_{ridge}$	0.999054	1.75* × *10^*−* 8^	6.93* × *10^*−* 5^
	${y}_{s}$	0.998446	6.46* × *10^*−* 8^	1.12* × *10^*−* 4^
4*(1.0, 1.3, 1.5, 1.3)	Depth	0.999648	1.1* × *10^*−* 9^	1.42* × *10^*−* 5^
	Period	0.995261	3.57* × *10^*−* 7^	3.10* × *10^*−* 4^
	${W}_{ridge}$	0.999004	1.85* × *10^*−* 8^	7.08* × *10^*−* 5^
	${y}_{s}$	0.998858	4.75* × *10^*−* 8^	9.80* × *10^*−* 5^
4*(1, 1, 1, 1)	Depth	0.999658	1.0* × *10^*−* 9^	1.297* × *10^*−* 5^
(without weights)	Period	0.995192	3.622* × *10^*−* 7^	3.122* × *10^*−* 4^
	${W}_{ridge}$	0.999117	1.64* × *10^*−* 8^	6.918* × *10^*−* 5^
	${y}_{s}$	0.998706	5.38* × *10^*−* 8^	9.98* × *10^*−* 5^

###  Optimized hyperparameters and ANN configuration

After conducting a Bayesian hyperparameter search using Optuna, the model arrived at a configuration that achieved the highest mean *R*^2^ across all cross-validation folds. The optimal settings included the number of hidden layers, the base number of neurons per layer, the dropout rate, the strength of L2 regularization, the choice of optimizer, the learning rate, and the mini-batch size as described in Table 4. This automated search process ensured that the final network structure and training parameters were chosen through a systematic exploration of the search space rather than being manually tuned. The best trial configuration reported by Optuna was then used to train the final proposed ANN model on the complete training set, establishing a solid baseline for future stacking and meta-learning steps.

**Table 4:** Standardized hyperparameter search space and the best values selected by Optuna.

Parameter	Search range	Best value (Optuna)
Batch size	[16, 128]	107
Number of layers	[2, 6]	3
Units per layer	[64, 512]	308
Dropout rate	[0.0, 0.5]	0.302
L2 regularization	10^*−* 7^to 10^*−* 3^	2.14* × *10^*−* 4^
Residual connection	{True, False}	True
Optimizers tested	Adam, Nadam, RMSprop	Adam
Max epochs	[0, 400]	Early stopping at epoch 57

### Model selection and final training

**Fig. 9:**
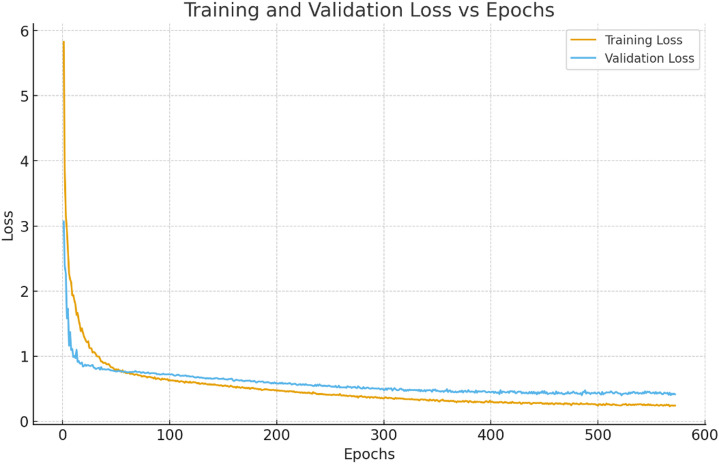
Training and validation loss curves of the proposed model training.

Early stopping was used to automatically restore the best-performing model weights. In this study, the minimum validation loss of approximately 0.2427 was achieved at epoch 572. This point, indicated by the red vertical dashed line in Fig. 9, was chosen as the “best epoch” for the final model weights. Training beyond this point did not lead to further improvements in validation performance, so it was not continued.

The predictive performance of the DNN at its optimal configuration was evaluated on the full dataset. Table 5 presents the *R*^2^, MSE, and mean absolute error (MAE) for each of the four target parameters. The ANN achieved *R*^2^ values of 0.999648 for ∆, 0.995261 for Λ, 0.999004 for ${W}_{ridge}$, and 0.998858 for ${y}_{s}$, all with correspondingly low MSE and MAE values. The overall multi-target *R*^2^ reached 0.998193, demonstrating the model’s ability to predict all four structural parameters with extremely high accuracy. All results in this study were performed using Google COOLAB CPU only, as GPU acceleration was not required for the proposed model size. Based on measured wall-clock time, the complete training pipeline—including preprocessing, polynomial feature expansion, Optuna hyperparameter search (30 trials), ANN training, and the stacking ensemble—required approximately 1 h of CPU runtime.

**Table 5:** Performance of the Optuna-optimized ANN at the best epoch (572).

Parameter	*R* ^2^	MSE	MAE	RMSE
Depth	0.999648	1.1* × *10^*−* 9^	1.42* × *10^*−* 5^	3.27531* × *10^*−* 5^
Period	0.995261	3.57* × *10^*−* 7^	3.10* × *10^*−* 4^	9.74370* × *10^*−* 5^
${W}_{ridge}$	0.999004	1.85* × *10^*−* 8^	7.08* × *10^*−* 5^	1.358806* × *10^*−* 5^
${y}_{slot}$	0.998858	4.75* × *10^*−* 8^	9.80* × *10^*−* 5^	2.178521* × *10^*−* 5^

## Results and performance evaluation of ANN and stacking ensemble

In this section, we present a comprehensive evaluation of the proposed ANN and stacking ensemble framework for the inverse design of the PPSBG sensor. We begin by examining the correlation between predicted and actual values using scatter plots to provide a visual measure of the model’s precision. then we describe the implementation workflow in detail, it shows how the dataset is generated, preprocessed, and used to train the ANN and stacking ensemble, and how the model outputs the corresponding geometric parameters for a given spectral input. Fabrication tolerance studies are then presented to evaluate the robustness of the predicted designs against realistic process variations. Finally, a literature comparison places the proposed method in the context of existing machine-learning-based approaches for photonic devices.

###  Scatter analysis

**Fig. 10:**
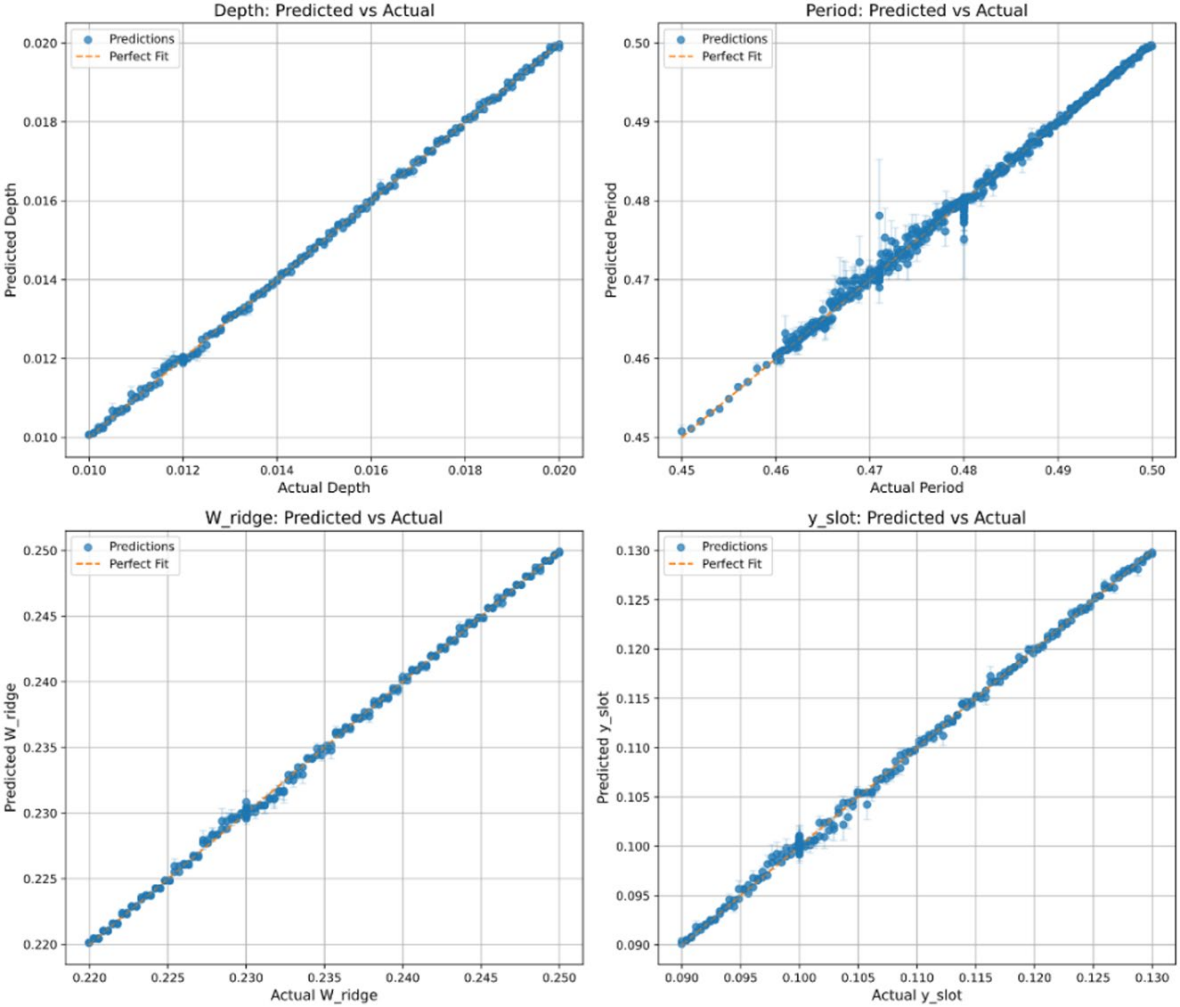
Scatter plots of actual versus predicted values for the PPSBG parameters.

The scatter plots of the actual versus predicted values for the four PPSBG structural parameters, as illustrated in Fig. 10, demonstrate a strong alignment of the data points along the diagonal reference line. The predictions corresponding to ∆, Λ, ${W}_{ridge}$, and ${y}_{s}$ exhibit close clustering around this line, indicating that the predicted values closely match the actual measurements. This strong correlation confirms the high accuracy and generalization capability of the developed ANN-based model for inverse design of the PPSBG structure.

###  Inverse-design framework workflow

**Fig. 11:**
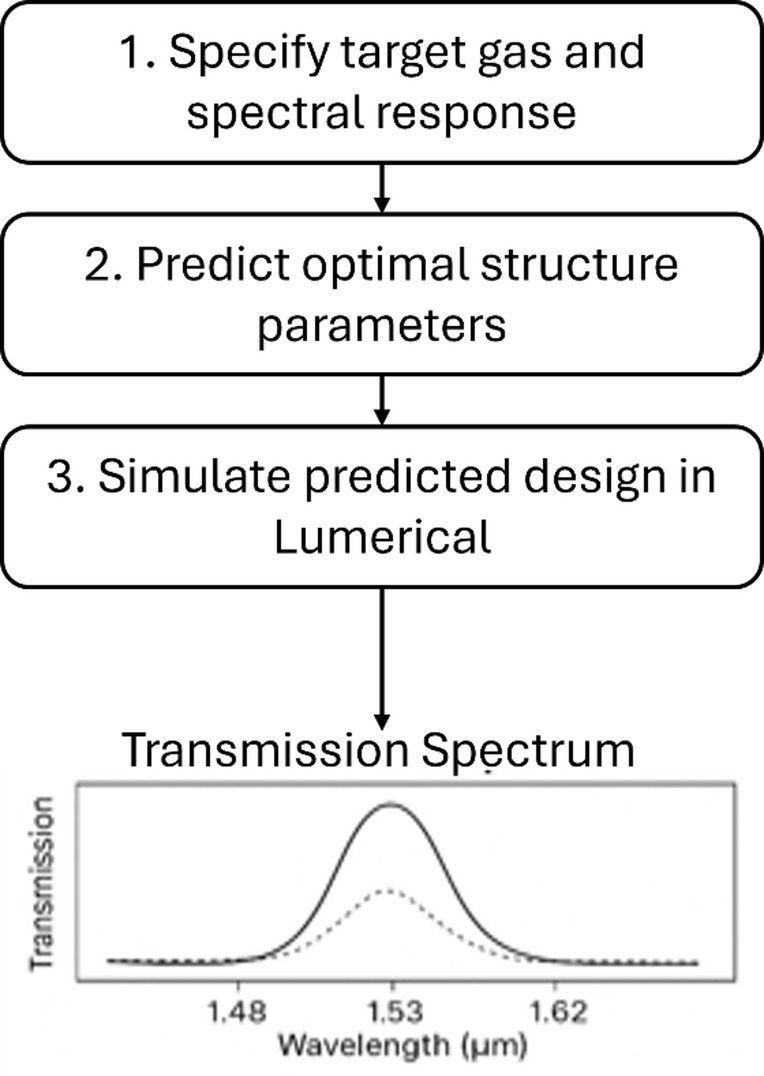
Scatter plots of actual versus predicted values for the PPSBG parameters.

The presents the workflow of the proposed inverse-design framework shows in Fig. 11. The process begins by allowing the user to specify the desired spectral characteristics of the target resonance. These spectral features are provided as inputs to the trained machine-learning model, then the model predicts the corresponding structural geometry of the PPSBG. The predicted dimensions are subsequently imported into the Lumerical FDTD simulator, where the Bragg grating structure is simulated and the transmission spectrum is obtained for verification.

### Evaluation using independent test scenario

**Fig. 12:**
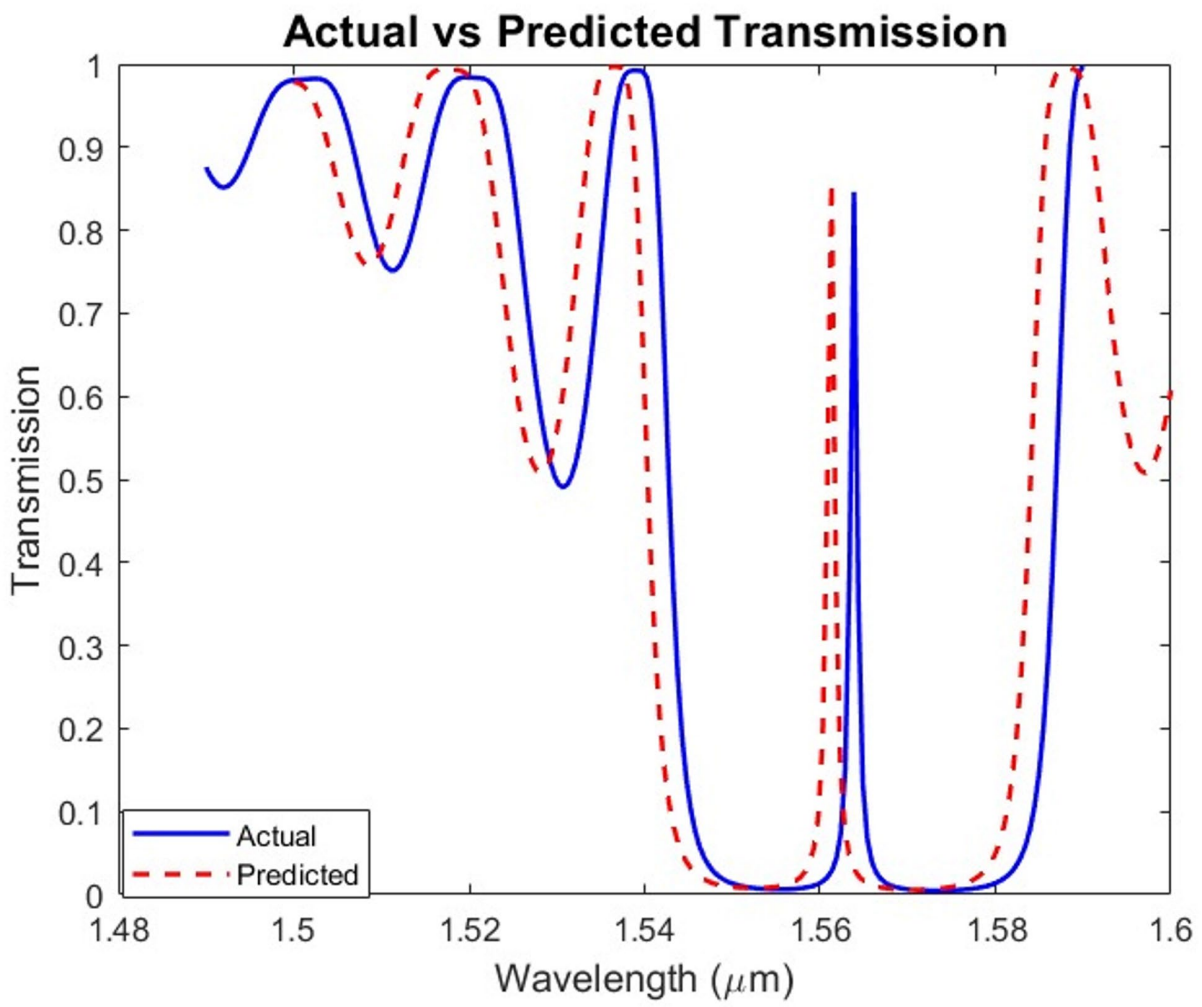
Actual versus predicted values transmission spectrum for testing data.

To evaluate the generalization capability of the proposed PPSBG inverse-design model, an unseen test case was conducted for $C{O}_{2}$ gas detection using a design that was not included in the training dataset. The proposed ML inverse-design framework achieves approximately 2000× speedup compared to conventional gradient-based iterative optimization, while maintaining over 99% spectral fidelity between the predicted structure and the target transmission response. The model was provided with input parameters corresponding to $C{O}_{2}$ gas, specifically a center wavelength of 1.55 *µ*m and the desired FWHM. Based on these inputs, the trained model predicted the structural parameters of the PPSBG as follows: ${\varLambda}_{pred}$= 0.48 *µ*m, ${W}_{ridge,pred}$= 0.23 *µ*m, ${d}_{pred}$ = 0.013 *µ*m, and ${y}_{s,pred}$= 0.093 *µ*m. These were compared with the actual optimized design parameters obtained from simulation, which are ${\varLambda}_{act}$ = 0.4709 *µ*m, ${W}_{ridge,act}=$ 0.245 *µ*m, ${d}_{act}$ = 0.014787 *µ*m, and ${y}_{s,act}$= 0.099 *µ*m. The close agreement between the predicted and actual values demonstrates the strong predictive accuracy of the developed model. As illustrated in Fig. 12, the transmission spectrum of the predicted structure exhibits an excellent match with the target optical response, confirming that the model effectively captures the nonlinear mapping between the spectral characteristics and the geometrical parameters of the PPSBG.

### Ablation study: contribution of ensemble base learners

**Fig. 13:**
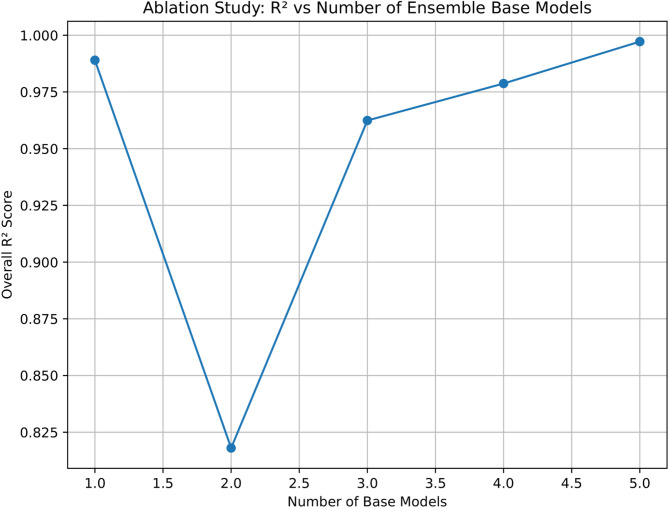
Ablation study illustrating the effect of increasing the number of base models in the stacking ensemble.

To evaluate the contribution of each learner in the proposed stacking architecture, we conducted an ablation study. This involved gradually increasing the number of base models from one to five and calculating the R² score for each configuration. As shown in Fig. 13, the performance of the ensemble consistently improves with the addition of more learners. Moving from one to three models shows an increase in the R² score, followed by steady improvements up to a full five-model setup. Importantly, we did not observe any decline in performance at any stage, which indicates that each added model provides valuable and complementary predictive information. The complete ensemble of five models achieves the highest R² value, confirming that this full configuration delivers the predictive implementation in the final architecture.

### Fabrication tolerance analysis

To address fabrication variability, a tolerance analysis was conducted by introducing *±* 5% and *±* 10% variations to the structural parameters of the designed PPSBG. The resulting transmission spectra were simulated and compared with the ideal response. The analysis shows that the PPSBG maintains stable resonance behavior for deviations up to *±*5 nm, with only minor shifts in the center wavelength and the peak resonance. At *±* 10 nm variations, the spectral response begins to degrade due to reduced modal confinement and mismatch between the Bragg condition and the effective index.

### Comparative analysis with existing literature

A comparative analysis of previous machine learning-based studies on Bragg gratings and the proposed PPSBG inverse design model is summarized in Table 6. Earlier research has shown the effectiveness of various learning architectures, such as XGBoost, multilayer perceptron (MLP) networks, and convolutional neural networks (CNNs), in improving the performance of optical sensors and filters. For instance, XGBoost demonstrated exceptional predictive accuracy for surface Bragg grating filters, while MLP-based ANNs have been successfully applied to biological cell detection using strip waveguide Bragg gratings. Additionally, regression-based machine learning frameworks for gold-coated tilted fiber Bragg gratings (FBGs) have enabled ultra-sensitive refractive index biosensing, and 1-D CNN models used with flexible dual FBG structures have exhibited high directional sensing accuracy. In contrast, the proposed PPSBG approach combines an Optuna-tuned ANN with a tree-based stacking ensemble, achieving robust and consistent performance with an *R*^2^ *>* 0.98 across all output parameters. Unlike previous studies that primarily focused on spectral analysis or deformation detection, this research emphasizes inverse structural design for gas sensing.

**Table 6:** Comparison of the proposed study with previous works on PPBG inverse design.

Study	Structure type	Model	Dataset size	Model accuracy	Application
^15^	Surface Bragg grating	XGBoost	800	0.999 (inverse de sign)	Optical filters
^23^	Strip waveguide Bragg grating	ANN (MLP classifier)	666 spectra	0.79 (classification)	Cancer cell detection
^24^	Gold-coated tilted fiber Bragg grating	Regression-based ML model	23,170 spectra (470exp.)	-	Real-time refractive index biosensing
^25^	Dual fiber Bragg grating (FBG)	1-D CNN	1,600 spectra	0.99 (classification)	Directional sensing for robotics and surgical ap plications
^26^	Chirped apodised fiber Bragg gratings	Deep learning	168 FBG spectra	-	Long-term structural health monitoring
^27^	Fiber Bragg grating	ML algorithms	2.7 × 10 distinct samples	0.99 (inverse de sign)	Overcoming inverse de sign problems
This work	Polymer phase-shift Bragg grating	Optuna-tuned ANN and tree-based stacking	1,045 designs	0.998 (inverse de sign)	Inverse design for gas sensing ($\mathrm{C}{\mathrm{O}}_{2}$, $\mathrm{C}{\mathrm{H}}_{4}$)

### Future work

Future work will focus on extending the proposed PPSBG-based sensing platform to additional material systems and broader spectral ranges. Since the design principles developed in this study are not limited to the silicon-on-insulator (SOI) platform, the device can be readily adapted to alternative photonic materials such as silicon nitride (Si_3_N_4_). This platform offers a wider transparency window, enabling operation from the visible to the near-infrared region. This flexibility would allow the sensor to target a wider range of gas species and concentrations by selecting appropriate polymer materials for the slot region, each tailored to specific refractive-index responses. In parallel, future investigations will incorporate experimentally measured data to further validate and refine the machine-learning inverse-design framework. The PPSBG structure designed in this work has already been submitted for fabrication, and the resulting prototypes will be characterized in terms of spectral response, gas sensitivity, and robustness to environmental variations.

## Conclusion

In this work, an inverse-design framework for a polymer phase-shift Bragg grating (PPSBG) sensor was developed using a hybrid deep learning model that combines an Optuna-tuned artificial neural network (ANN) with a tree-based stacking ensemble. The model can learned the nonlinear relationships between spectral characteristics and structural parameters across 1045 design samples, achieving a high accuracy with *R*^2^ *>* 0.98 for all predicted outputs. The proposed model was validated using unseen test data, where the model was tasked with generating a new grating design for CO_2_ gas detection. The resulting structure, when simulated in the Lumerical simulator, produced a transmission spectrum that closely matched the target spectral response, confirming the model’s reliability and physical interpretability. The outcomes of this study demonstrate the strong potential of deep learning–driven inverse design for accelerating the development of high-performance photonic sensors. By bridging data-driven prediction and physical simulation, the proposed method enables efficient design exploration and optimization for complex photonic structures. Future work will focus on extending this approach to multi-gas detection, incorporating fabrication tolerances.

.

## Data Availability

The datasets used and/or analyzed during the current study are available from the corresponding author on reasonable request.
